# Identification and Validation of miRNAs as Endogenous Controls for RQ-PCR in Blood Specimens for Breast Cancer Studies

**DOI:** 10.1371/journal.pone.0083718

**Published:** 2013-12-31

**Authors:** Ailbhe M. McDermott, Michael J. Kerin, Nicola Miller

**Affiliations:** Discipline of Surgery, School of Medicine, National University of Ireland, Galway, Ireland; University of Alabama at Birmingham, United States of America

## Abstract

**Introduction:**

A prerequisite to accurate interpretation of RQ-PCR data is robust data normalization. A commonly used method is to compare the cycle threshold (C_T_) of target miRNAs with those of a stably expressed endogenous (EC) miRNA(s) from the same sample. Despite the large number of studies reporting miRNA expression patterns, comparatively few appropriate ECs have been reported thus far. The purpose of this study was to identify stably expressed miRNAs with which to normalize RQ-PCR data derived from human blood specimens.

**Methods:**

MiRNA profiling of approximately 380 miRNAs was performed on RNA derived from blood specimens from 10 women with breast cancer and 10 matched controls. Analysis of mean expression values across the dataset (GME) identified stably expressed candidates. Additional candidates were selected from the literature and analyzed by the geNorm algorithm. Further validation of three candidate ECs by RQ-PCR was performed in a larger cohort (n = 40 cancer, n = 20 control) was performed, including analysis by geNorm and NormFinder algorithms.

**Results:**

Microarray screening identified 10 candidate ECs with expression patterns closest to the global mean. Geometric averaging of candidate ECs from the literature using geNorm identified *miR-425* as the most stably expressed miRNA. *MiR-425* and *miR-16* were the best combination, achieving the lowest V-value of 0.185. Further validation by RQ-PCR confirmed that *miR-16* and *miR-425* were the most stably expressed ECs overall. Their combined use to normalize expression data enabled the detection of altered target miRNA expression that reliably differentiated between cancers and controls in human blood specimens.

**Conclusion:**

This study identified that the combined use of 2 miRNAs, (*miR-16* and *miR-425*) to normalize RQ-PCR data generated more reliable results than using either miRNA alone, or use of *U6*. Further investigation into suitable ECs for use in miRNA RQ-PCR studies is warranted.

## Introduction

Accumulating evidence has shown that miRNAs play pivotal roles in regulatory functions pertaining to cell growth, development and differentiation and are associated with a wide variety of human diseases. Despite their discovery over a decade ago, it is only recently that the extent of the complexity of these regulatory molecules is beginning to be understood. Expression analysis studies have revealed differential miRNA expression in tumors compared to normal tissues. MiRNAs have been found to be dysregulated in a wide variety of human cancers. Accordingly, miRNAs have elicited much interest as biomarkers for cancer diagnosis and disease monitoring and are rapidly emerging as novel targets for disease intervention.

Real-time quantitative (RQ-PCR) is widely used to quantify miRNA expression due to its sensitivity, specificity, speed, simplicity and the small amounts of template RNA required. To differentiate true biological variation from experimentally induced artifacts, target miRNA expression levels are normalised to those of a control(s). To accurately quantify miRNA expression by RQ-PCR, samples are assayed during the exponential phase of the PCR reaction during which time the amount of target miRNA is presumed to double with each cycle, without influence from limiting reagents. Comparison of cycle threshold (C_T_), the cycle number at which fluorescence signals are detected above background, to C_T_ values to an endogenously expressed control RNA is used to determine miRNA expression levels by relative quantification (EC). The accuracy of this method is heavily reliant on the choice of endogenous control. Other methods of normalization such as normalization to the global mean, use of spike-in controls, among others have also been described [1]. Regardless of the approach, the normalization technique and specific control RNA(s) used directly influence the results produced from RQ-PCR and thus validity. The selection of a suitable EC(s), with which to normalize RQ-PCR data, is an important first step in the accurate and reliable determination of miRNA expression levels.

Ideally a reliable EC(s) should remain stably expressed regardless of disease status or other clinical variables. A set of robust ECs that are steadily uniformly expressed across all body tissues, fluids and disease pathways has yet to be described, and is unlikely to exist. Several miRNA expression analysis studies based on tissue have reported the use of small RNAs (such as *U6, RNU44* or *RNU48*) or *miR-16* to normalize expression data [2]–[6]. However, use of these reference genes cannot simply be applied to miRNA analysis in blood or other body fluids as miRNA expression patterns are known to be disease-specific and perhaps specimen-type specific [7]. Despite the abundance of studies on circulating miRNA profiles to discriminate between normal and disease states, there have yet to be conclusive reports of appropriate ECs. This remains a significant hurdle that must be addressed to substantiate biomarker discovery and validate single miRNA expression analysis using RQ-PCR.

Breast cancer is a prevalent disease with increasing incidence worldwide. This growing social and economic burden has stimulated the search for novel biomarkers to aid in diagnostics, prognostication and disease monitoring of adjuvant treatment. Few validated endogenous controls for miRNA research in breast cancer have been described. Initial miRNA studies on breast tissues by Mattie *et al* normalized miRNA expression to *miR-16* and *let-7*, which were later shown to be stably expressed across malignant, benign and normal breast tissue by Davoren *et al* [2], [8]. Early studies on systemic miRNAs in breast cancer normalized to *miR-16* [9], [10]. Additional studies, on breast and other cancers, have suggested alternative EC candidates such as *U6, RNU44, RNU48*, *miR-142-3p*, *miR-484*, *miR-191* and *miR-425* [3], [11]-[18]. However, there is a lack of validated reports of suitable ECs for circulating miRNAs.

The aims of this study were to evaluate a panel of candidate ECs (using microarray profiling) from which to validate the most stably expressed EC(s) to normalize RQ-PCR data derived from blood specimens in breast cancer. In addition we wished to determine the effect of different normalization strategies on target miRNA expression.

## Materials and Methods

### Study cohort and sample collection

Blood samples were prospectively collected from 80 women including 50 consecutive patients with a new diagnosis of breast cancer and 30 healthy control participants. All patients had histologically confirmed invasive breast cancer. Samples of venous non-fasting blood were collected in BD vacutainers containing 18 mg dipotassium EDTA (K2E) anticoagulant (BD-Plymouth, PL6 7BP, UK) following written informed consent. Microarray profiling was performed on RNA derived from blood on 10 of the above patients and 10 of the controls (Table 1). The remaining 40 cases and 20 controls were used to validate candidate ECs and target miRNA expression (Table 1). Ethical approval was granted by the Clinical Research Ethics Committee, Galway University Hospital, Ireland.

**Table 1:** Clinico-pathological data for blood samples derived from breast cancer cases and controls for microarray and RQ-PCR analysis.

Tumors	Array	RQ-PCR
	Number (%)	Number (%)
	10	40
Mean age, years (range)	56.7	56.17
Median whole. T size (mm)	45.6 (±31.27)	30.55 (±19.47)
Missing data	-	2 (5)
Nodal status		
Positive	5 (50)	20 (50)
Negative	5 (50)	20 (50)
Grade		
1	1 (10)	7 (17.5)
2	9 (90)	25 (62.5)
3	-	8 (20)
UICC stage		
Stage 1	2 (20)	15 (37.5)
Stage 2	5 (50)	12 (30)
Stage 3	3 (30)	12 (30)
Missing	-	1 (2.5)
Intrinsic Subtype		
Luminal A	10 (100)	30 (75)
Luminal B	-	2 (5)
HER2/*neu*	-	5 (12.5)
Basal	-	3 (7.5)
**Controls**	**10**	**20**
Mean Age, years (range)	81.7	49.65

Mm, millimeter; UICC, breast tumor staging according to the International Union Against Cancer criteria; HER2/*neu*, human epidermal growth factor receptor. Control subjects had no personal or family history of breast or ovarian cancer and were clinically well at the time of sampling.

### RNA extraction and analysis

Total RNA was extracted from 1 mL of blood using TRI Reagent BD (Molecular Research Centre, Inc). RNA concentration and integrity were examined by NanoDrop spectrophotometry (NanoDrop ND-1000 Technologies Inc., DE, USA) and Agilent Bioanalyzer RNA 6000 Nano Chip Kit Series II (Agilent Technologies, Germany) analysis, respectively.

### Microarray profiling

Expression profiling of circulating miRNAs was performed on RNA extracted from 20 blood specimens using TaqMan human miRNA microarrays as instructed by the manufacturer (TaqMan Low Density Array Human microRNA Card A, Life Technologies, Foster City, CA, USA). Megaplex primer pools were used to reverse transcribe RNA samples (700 ng) which were then PCR amplified in 2 µL volumes on 384 well pre-configured microfluidic cards. Each card contained TaqMan probes for 377 miRNAs plus 3 pre-defined ECs (*U6* in quadruplicate, *RNU44* and *RNU48*) and a negative control (*ath-miR-159a*).

### Candidate EC selection

In addition to the candidate ECs identified by microarray profiling, the expression of 7 additional candidates, as chosen from a review of published studies, was investigated in the array dataset (*miR-16, miR-425, miR-484, miR-142-3p U6, RNU44* and *RNU48*, Table 2). Three of these (*miR-16, U6*, and *miR-425*) were further validated in a larger cohort of blood from breast cancer patients and controls.

**Table 2:** Candidate endogenous controls.

MiRNA Name	Molecule type	Accession Number*	Chromosomal Location	Blood Component	Reference
miR-16	miRNA	MI0000070*	13q14.2	Whole blood, Serum, Plasma	[9], [14], [25], [30]-[34], [49]
U6 (RNU6B)	snoRNA	26826**	10p13	Plasma	[11], [23]
RNU48	snoRNA	26801**	6p21.33	Serum	[50]
miR-425	miRNA	MI0001448*	3p21.31	Whole blood	[17]
RNU44	snoRNA	26806**	1q25.1	Whole blood	[51]
miR-484	miRNA	MI0002468*	16p13.11	Serum	[15]
miR-142-3p	miRNA	MI0000458*	17q22	Plasma	[14]

mirBase database accession number

NCBI Gene ID

### Data analysis

Microarray data was analyzed in two ways. Firstly, to identify the most stably expressed miRNA(s) from the panel of 377 miRNAs across the 20 blood samples, global mean expression normalization was applied [19]. MiRNAs were then ranked commencing with those with expression profiles closest to that of the mean. The second approach used the GeNorm algorithm to assess the stability of the 7 candidate ECs chosen from the literature. The most stably expressed ECs were assessed and ranked both individually (M variable) and in combination (V variable) [20].

### RQ-PCR validation

Total RNA (100 ng) was reverse transcribed using miR-specific stem-loop RT primers (Life Technologies) and components of the High Capacity cDNA Reverse Transcription kit (Invitrogen Life Technologies) according to the manufacturer's protocols. Expression levels of individual miRNAs were detected by subsequent RQ-PCR using TaqMan MicroRNA assays (Invitrogen Life Technologies) and a 7900HT instrument (Life Technologies) using standard thermal cycling conditions in accordance with manufacturer recommendations. PCR reactions were performed in triplicate in final volumes of 10 µl on 96 well plates, including inter-assay controls (IAC) to account for variations between runs.

### Amplification efficiency

PCR amplification efficiencies (E) were generated using the formula E = (10-1/slope - 1)×100, using the slope of the semi-log regression plot of C_T_ versus log input of cDNA (10-fold dilution series of five points). A threshold of 10% above or below 100% was adopted.

### PCR data analysis

Cycle threshold (C_T_), or quantification cycle (Cq) is the cycle number during a PCR reaction at which the fluorescence generated is sufficient to pass the threshold, ten times the standard deviation of the baseline fluorescence emission. C_T_ values inversely correlate with the logarithm of the initial expression such that candidates with high expression had low C_T_, and vice versa. The threshold standard deviation for intra- and inter-assay replicates was 0.28.

### Candidate EC stability analysis

Stability of candidate ECs was assessed using geNorm and NormFinder algorithms. GeNorm is based on the assumption that none of the candidate ECs is co-regulated. It was used to rank ECs according to stability values (M), which represented the variation in expression of candidate ECs in comparison to each other. This was done by selecting optimal pairs of ECs by calculating and comparing M values for all candidates and stepwise exclusion of the least stable EC [20]. NormFinder is a Microsoft Excel add-in that can accommodate both inter- and intra-group variation [21], in this case cancer and control, by accounting for variability and bias between groups. It was used to estimate the most stable EC in isolation, and the most stable 2-EC combination. The lower the stability value, the more stable the expression of the EC candidate.

### Comparative quantification of target miRNAs relative to EC

Target miRNA (*miR-93, miR-181a* and *miR-652*) expression levels were estimated using qBase software (Biogazelle, Belgium) to calculate amplification efficiency-corrected relative quantities following normalization to each candidate EC. The comparative cycle threshold (ΔΔC_T_) method, using the formula ΔΔC_T_ = (C_T_ target, test sample - C_T_ EC, test sample) – (C_T_ target, calibrator sample - C_T_ EC, calibrator sample) was applied. To test the effect of alternative EC on target miRNA detection, the expression of miRNA targets (*miR-181a, miR-652* and *miR-93*) with previously documented expression in the circulation of breast cancer patients were measured using the ECs.

### Statistical analysis

Statistical analysis was performed using Minitab version 16.0 (Minitab Ltd, Coventry UK). The Kolmogorov-Smirnov test for normality was conducted and parametric tests were used where appropriate. A log transformation (log_10_) of the data was performed when necessary. Significance of circulating miRNA levels was determined using t-tests or Mann-Whitney U test, as appropriate. Results with p values <0.05 were considered significant.

## Results

### Identification of candidate ECs (using microarray)

To identify the most stably expressed miRNAs from the microarray dataset global mean expression normalization was applied. This involved the use of the mean expression value of all expressed miRNAs in a given sample (in this case, 20 samples) as a normalization factor for miRNA RQ-PCR. MiRNAs with expression profiles closest to the mean were *miR-103, miR-185, miR-532-3p*, *miR-194, miR-126, miR-155, let-7e, miR-345, miR-425 and miR-15b* as illustrated in Table 3. The first 9 miRNAs on this list were excluded from further EC analysis on the basis of their documented roles in breast cancer (Table 3). *MiR-425* was the only miRNA in this group not previously implicated in breast cancer and hence was chosen for further validation by RQ-PCR.

**Table 3:** GME analysis to determine 10 most stably expressed miRNAs from the microarray dataset.

Rank	miRNA	Previous Reports	Standard Deviation from GME	Reference
1	miR-103	Upregulated in serum of breast cancer patients	0.121	[52], [53]
2	miR-185	Suppress tumor growth and progression in human ovarian cancers, pediatric renal tumors and breast cancer cell lines	0.128	[54]
3	miR-532	Associated with triple negative(ER-ve/PR-ve/HER2/neu-ve) breast cancer in tumor tissue	0.132	[55]
4	miR-194	Upregulated following Traztuzumab therapy in breast cancer cells; overexpression of miR-194 results in cell migration and invasion inhibition in breast cancer cell lines	0.139	[56]
5	miR-126	Under-expressed in breast cancer, with restoration associated with metastases suppression in breast cancer cell lines and breast tumors	0.150	[57]-[59]
6	miR-155	Over-expressed in breast tissue and circulation of women with breast cancer	0.155	[60], [61]
7	let-7c	Under-expressed in breast cancer with a tumor suppressor role	0.158	[9], [62], [63]
8	miR-345	Targets MRP in multidrug resistant breast cancer cells compared to normal cells	0.158	[64]
9	miR-425	No reports of altered expression or functional role in breast cancer	0.160	-
10	miR-15b	Altered expression in circulation and tumor of those with breast cancer	0.162	McDermott *et al*., unpublished data

The top 10 most stably expressed miRNAs following normalization of the microarray data using global mean expression (GME). Nine of these miRNAs have been implicated in breast cancer. *MiR-425* is the only candidate miRNA with no reported association with breast or any other cancer type.

**Figure 1:**
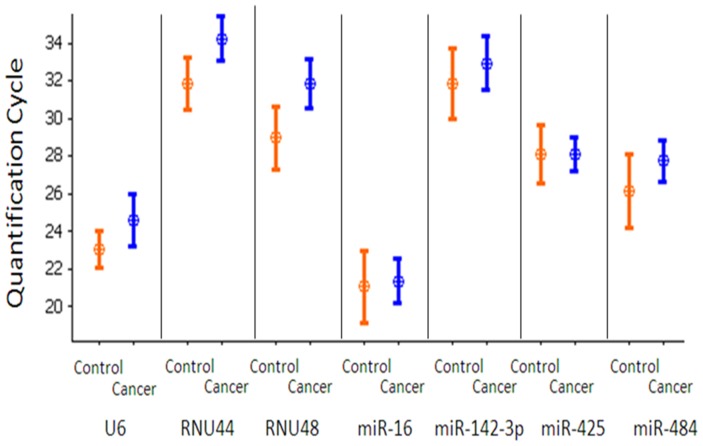
Quantity of each candidate miRNA on microarray analysis. The quantity of each miRNA or snoRNA (quantification cycle/cycle threshold) was determined by microarray for the cancer group and the control group. There was no significant difference (t-test) in candidate EC expression between the cancer group and the control group.

**Figure 2:**
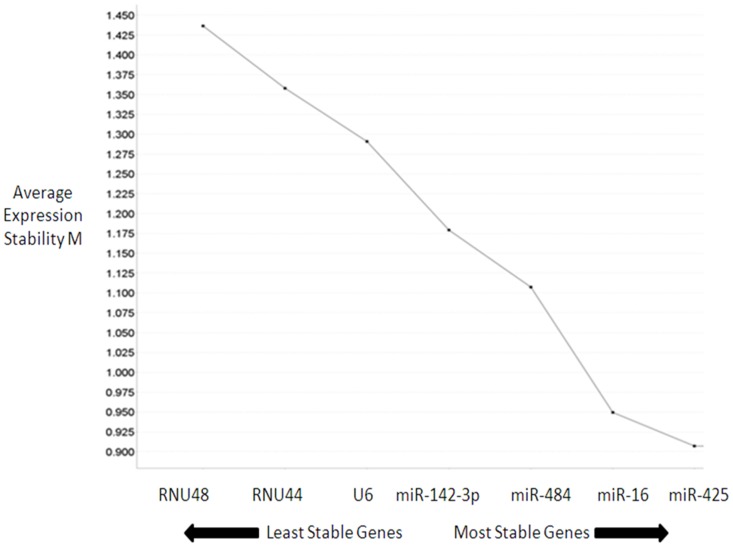
GeNorm analysis of average expression stability of candidate ECs. Ranking of candidate ECs according to average expression stability. The least stable candidate ECs with the highest stability measure (M) are on the left side of the graph, with the most stable ECs with the lowest M value on the right. *RNU48* and *RNU44* are the least stable ECs while *miR-425* and *miR-16* are the most stable candidate ECs.

**Figure 3:**
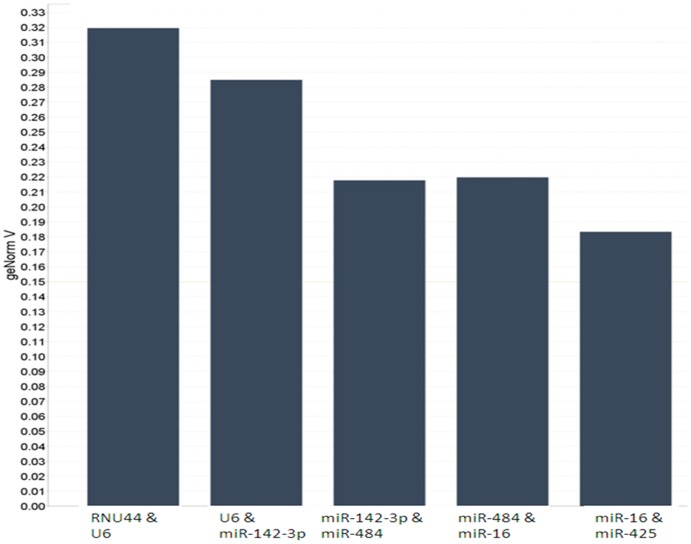
Determination of the best combination of ECs for normalization. Determination of optimum number of candidate ECs for normalization. The GeNorm programme establishes the optimum combination of candidate ECs for normalization, producing the lowest V variable. This factor is calculated using the variable ‘V’ as the pairwise variation (Vn/Vn+1) between two sequential normalization factors (NFs) (NF_n_ and NF_n+1_). The combination of candidate ECs is deemed optimal when the V variable achieves the lowest value. The optimal combination was achieved by combining *miR-16* and *miR-425*.

There was no significant difference in expression of the candidate miRNAs and snoRNAs between the cancer and control group across the microarray dataset (Figure 1). Expression stability of the means of snoRNAS (*U6, RNU44* and *RNU48*) and miRNAs (*miR-16*, *miR-425*, *miR-142-3p*, and *miR-484*) were assessed using GeNorm (Figure 2). *MiR-425* was found to be the most stably expressed, with a geNorm M-value 0.907. RNU48 was the least stably expressed candidate. Combination of *miR-425* and *miR-16* resulted in the lowest V- value of 0.185 (Figure 3).

The stability of 3 of the above miRNAs (*miR-425*, *miR-16* and *U6*) was further investigated by RQ-PCR in a larger cohort: *miR-425* was selected as it was identified by GME analysis and had the lowest geNorm M value. *MiR-16* had the next lowest geNorm M value and has been used to normalize PCR data in several cancer studies both in tissue and blood [8], [9], [14], [22]. *U6* was selected, not on the basis of its apparent stability (it did not feature in GME analysis, and ranked comparatively low by geNorm), rather due to its wide use in the literature [11], [23].

### Relative quantities of candidate EC genes

RQ-PCR was performed to validate the expression patterns of 3 candidate ECs in 60 blood samples, comprised of 40 samples from women with cancer and 20 from healthy controls (2). All candidate ECs were expressed in abundance, with mean C_T_ values less than 25. *MiR-16* showed the highest expression, with mean C_T_ of 15.5 (range 13.5–18.7), followed by *miR-425*, mean C_T_ 20.7 (range 17.4–24.2) and then *U6*, mean C_T_ 21.0 (range 19.0–22.8), see Table 4.

**Table 4:** Cycle Threshold (C_T_) values for ECs in validation cohort.

miRNA	Mean C_T_ ± St Dev	C_T_ Range	C_T_ Min	C_T_ Max
U6	21.042±0.848	3.843	19.065	22.898
Cancer	21.23±0.854		19.568	22.898
Control	20.661±0.712		19.065	21.804
miR-16	15.460±1.342	5.2	13.565	18.765
Cancer	15.398±1.346		13.565	18.765
Control	15.584±1.361		13.585	17.812
miR-425	20.740±1.415	6.746	17.459	24.206
Cancer	20.772±1.416		18.100	24.206
Control	20.676±1.447		17.459	23.395

**Figure 4:**
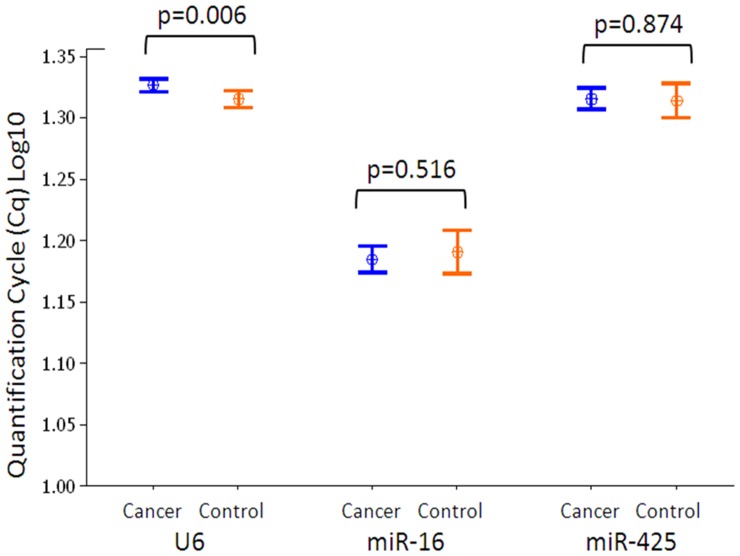
Relative quantity candidate ECs. Relative quantity of candidate EC miRNAs in blood of breast cancer patients (blue, n = 40) and healthy controls (n = 20) expressed as quantification cycle (Cq) values. Interval plots display the mean and 95% confidence interval. There was no significant difference (p>0.05, t-test) for *miR-16* and *miR-425*. However, *U6* was more abundant in the control group (p = 0.006).

The C_T_ values of each candidate EC were assessed for differential expression between cancer and control blood samples (Figure 4). *U6* was significantly more abundantly expressed in the control group (p = 0.009). In this manner it was identified that there was no difference in expression of *miR-16* or *miR-425* between the cancer group and the controls, as would be expected for good candidate ECs.

**Figure 5:**
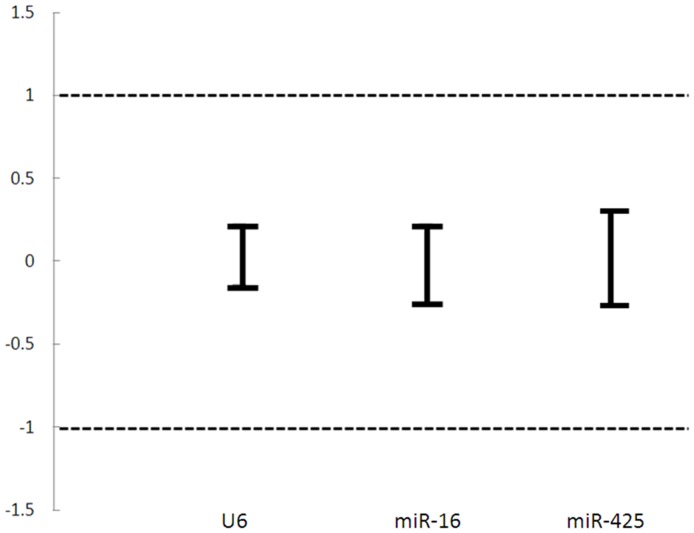
Equivalence test for candidate ECs. Each line represents the difference in logarithmic (log base2) expression between the cancer and control groups. The upper and lower bars of individual candidate ECs represents the upper and lower limits of symmetrical confidence intervals, respectively. Confidence intervals between −1 and +1 corresponded to fold changes of ≤2. No candidate EC displayed a fold change greater than 2. All three candidate ECs were equivalently expressed.

Relative expression values of candidate ECs were log transformed and expressed as means with matching symmetrical confidence intervals (CI). Confidence intervals between -1 and +1 represented fold changes of ≤2, while those between −1.58 and +1.58 equated to fold changes of ≤3. A fold change cut off of 3 was applied as previously established [24]. Confidence intervals with an upper border >1.58 signaled over-expression of a candidate EC in the cancer group. Confidence intervals with lower borders <1.58 indicated under-expression of the candidate EC in the control group (Figure 5). Equivalence testing was then performed to confirm that all three candidate ECs were equivalently expressed between the cancer group and the control group.

**Figure 6:**
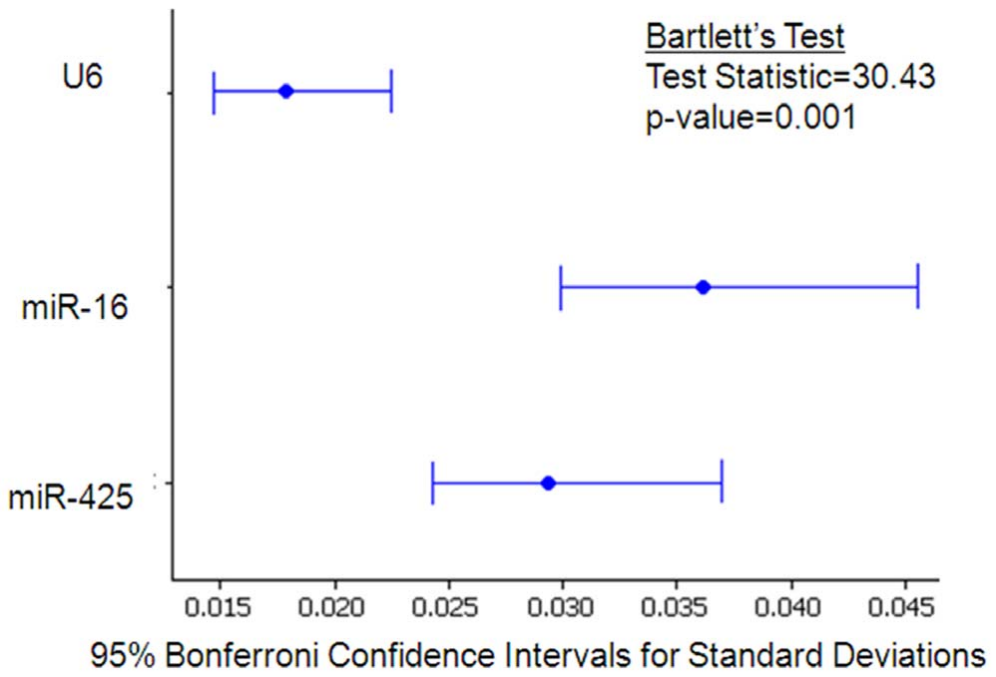
Variation associated with each candidate EC. Bonferroni confidence intervals for standard deviations. There was a significant difference in variance (p<0.001, Bartletts's test) associated with each candidate EC, indicating differing stabilities.*MiR-16* showed greater variance than *miR-425* and *U6*.

There was a significant difference in variance between ECs (Bartlett's test, p<0.001) indicating their differing stabilities, with *miR-16* showing the greatest variation (Figure 6).

### Analysis of reference gene expression stability geNorm and NormFinder

The stability and variability of the candidate ECs was further assessed using NormFinder and geNorm as summarized in Table 5. Lower stability values indicate greater stability. GeNorm provided two values: a gene stability (geNorm M) value and a geNorm V value. The geNorm M value ranked candidate ECs according to their stability, from the least stable (highest M value) to the most stable candidate (lowest M value). These values were generated on the basis of the average pairwise variation between all tested genes accompanied by stepwise exclusion of the least stable gene. GeNorm V values determined the optimum EC pairing for normalization, by defining the pairwise variation between two sequential normalization factors. GeNorm identified *miR-16* as the single most stably expressed miRNA, with a GeNorm M value of 1.191. NormFinder identified *miR-16* and *U6* as the best combination, with a stability value of 0.102. The single best EC as calculated by this algorithm was *miR-425,* followed by *miR-16* and *U6*. Consistent with the geNorm analysis on the microarray data *miR-16* and *miR-425* were identified as being the most appropriate ECs.

**Table 5:** GeNorm and NormFinder expression stability analysis.

Rank	geNorm		NormFinder	
	Gene	Stability	Gene	Stability (M)
1	miR-16	1.191	miR-425	0.038
2	U6	1.232	miR-16	0.064
3	miR-425	1.251	U6	0.067

Ranking of candidate reference genes based on expression stability values calculated by NormFinder and geNorm

### Effect of candidate EC selection for normalization on relative expression of target miRNAs

**Figure 7:**
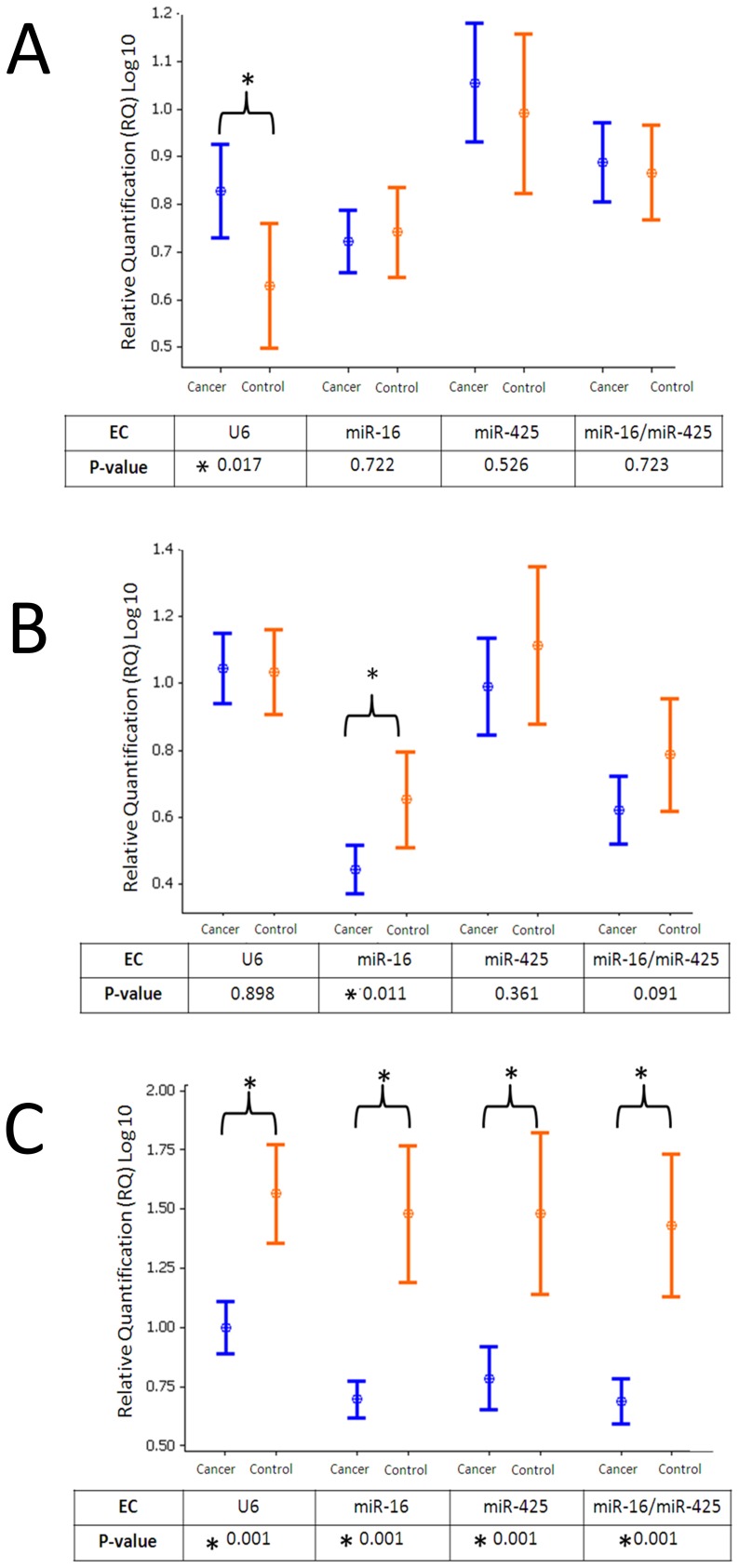
Effect of candidate EC selection on relative expression of target miRNAs. This figure demonstrates the impact of candidate EC selection on the accuracy of target miRNA expression. The relative expression of three target miRNAs (*miR-93, miR-181a* and *miR-652*) is presented following normalization using each of four distinct EC strategies: *U6* alone, *miR-16* alone, *miR-425* alone and finally *miR-16* and *miR-425* in combination. Interval plots depict mean and 95% confidence intervals for relative *miRNA* expression (Log_10_) in the blood of women with breast cancer (blue) and healthy controls (orange) normalized to different candidate ECs with p-values indicated in the table below. A. *MiR-93* expression. *MiR-93* appears to be elevated in the circulation of women with breast cancer when *U6* was used to normalise RQ data. However, when the other EC candidates are used for normalization there is no difference in *miR-93* expression between the cancer and control group. B. *MiR-181a* expression. *MiR-181a* is underexpressed with *mIR-16* was used to normalise RQ data (p = 0.011). There is no difference *in miR-181a* expression when other EC candidates are used for normalization. C. *MiR-652*. *MiR-652* is under-expressed in the circulation of women with breast cancer, regardless of the choice of candidate EC (*U6*, *miR-425* or *miR-16*) indicating that it was highly differentially expressed in blood of those with breast cancer.

To test their efficacy on target miRNA quantification, each of the candidate ECs was used to determine relative quantities of known breast cancer-associated miRNAs (Figure 7). *MiR-93*, which was previously shown not to be dysregulated in breast cancer (McDermott *et al*., unpublished data) was overexpressed in the cancer group when *U6* (p = 0.017) was used as an EC but was unaltered with other candidate ECs. *MiR-181a*, which was previously shown to be under-expressed in breast cancer [25](McDermott *et al*., unpublished data) was under-expressed when *miR-16* (p = 0.011) used as EC. Of the four target miRNAs, the choice of EC did not influence the relative quantity of circulating *miR-652* between cancers and controls (p<0.001) suggesting a highly significant differential expression of *miR-652* in breast cancer (McDermott *et al*., unpublished data). Relative quantities of target miRNAs in cancer and control groups are shown in Figure 7.

These results highlight the importance of selecting appropriate and validated ECs. Despite the large sample size, true biological differences in miRNA expression were not detected when using less stable ECs for normalization.

## Discussion

Altered miRNA expression is associated with most pathological disease processes, including carcinogenesis. Their ease of detection in biological fluids, including blood, makes them ideal candidates for exploitation as minimally invasive biomarkers. RQ-PCR is the most common technique for miRNA expression analysis. However, the high sensitivity of this approach means that accurate interpretation of RQ-PCR results depends heavily on the use of suitable, stably expressed ECs for data normalization in an effort to minimize non-biological variation between samples. Reference genes for mRNA studies have been well established but validated ECs for miRNA research are scarce. In addition, ECs for use in tissue miRNA research may not be directly translated to other tissues or body fluids. Scrupulous miRNA data normalization may be more important than other functional RNA classes [26].

The first systematic assessment of candidate ECs for miRNA RQ-PCR was conducted by Davoren *et al* [8]. This study examined the expression stability of five miRNAs (*let-7a, miR-10b, miR-16*, *miR-21* and *miR-26b*) and 3 small nucleolar RNAs (*RNU19, RNU48* and *Z30*) was determined in normal, benign and malignant breast tissue. The best normalization strategy for miRNA analysis in breast tissue was found to be a combination of *miR-16* and *let-7a*. There have been subsequent isolated reports of suitable ECs for specific disease states and specimen types, but the focus of this issue in the literature is disproportionate to the number of accounts of altered miRNA expression in specific disease states [15], [27]–[29]. Chang *et al* conducted a similar systematic approach to identify suitable ECs for application to colorectal cancer tissue [28]. MiRNA profiling was performed on a small cohort of paired colorectal tumor tissues and normal tissue. Global mean expression analysis was performed to identify stably expressed candidate ECs. Six candidate miRNAs (*let-7a*, *miR-16*, *miR-26a*, *miR-345*, *miR-425* and *miR-454*) and 2 small nucleolar RNAs (*RNU48* and *Z30*) were chosen for for further validation by RQ-PCR in a larger cohort of colorectal tissues. *MiR-16* and *miR-345* were identified as the best combination of reference miRNAs by both geNorm and NormFinder, with *miR-16* and *miR-345* being the single best normlizers identified by NormFinder and geNorm, respectively. Genovesi *et al* identified ECs for use in medulloblastoma studies involving TLDA cards, and recommended the combination of *miR-301a* and *miR-339-5p* for normalization of card A data, with a combination of miR-425* and *RNU24* being used for Card B data analysis [27]. Few studies have examined suitable ECs for use in circulating miRNA studies. Hu *et al* identified and validated candidate miRNAs as ECs for serum miRNA expression studies in breast cancer [15]. In this cohort, a combination of *miR-191* and *miR-484* provided the best normalization approach for target miRNA expression. Song *et al* focused on gastric cancer, examining 6 miRNAs (*let-7a, miR-16, miR-93, miR-103, miR-192, and miR-451*) and one small nucleolar RNA, *RNU6B* for suitability as candidate ECs [29]. This study advocated the use of *miR-16* and *miR-93*, the most stably expressed candidate ECs, for normalization of miRNA expression in serum for gastric cancer.

The present study identified that the combined use 2 miRNAs, (*miR-16* and *miR-425*) to normalize RQ-PCR data generated more reliable results than using either miRNA alone, or use of *U6*, which has been used by several authors to date. In the absence of a comprehensive analysis of reliable ECs for RQ-PCR data from blood samples, a microarray screen was performed at the outset. We profiled 20 blood samples (10 from women with breast cancer and 10 from healthy control women) for the expression of in excess of 380 miRNAs (including *U6* rRNA). The dataset was analyzed using global mean expression (GME) to identify miRNAs with expression patterns closed to the mean expression of the entire dataset. We selected *miR-425* from the GME analysis, and both *miR-16* and *U6* from the literature for further analysis by RQ-PCR in a validation cohort (n = 60). Our initial validation step using raw C_T_ values of these 3 candidate ECs displayed that *U6* was more abundant in the control group, while there was no difference in *miR-16* or *miR-425* expression between the cancer or control group. Equivalent expression of candidate ECs between the cancer and control group was then confirmed using a fold change cut off of ≤3, corresponding to confidence intervals between −1.58 and +1.58. We then used both GeNorm and NormFinder algorithms which identified *miR-16* and *miR-425*, respectively, as the most stably expressed candidate ECs, with NormFinder suggesting their combination as the best combination.

As evident from the results presented in this study, use of an inappropriate EC for normalization can significantly alter the apparent expression of target miRNAs. Combination of *miR-16* and *miR-425* as EC detected significant dysregulation of *miR-652* (p = 0.001). *MiR-181a* has previously been shown to be under-expressed in breast cancer, but was shown in this study to be significantly under-expressed when *miR-16* alone was used as an EC. Normalization with the combination of *miR-16* and *miR-425* increased the p-value to 0.091. *MiR-93* has been shown to be stably expressed in blood of women with breast cancer compared to healthy controls. However, when *U6* was applied as the normalizer *miR-93* appeared to be upregulated in the cancer group. A combination of miRNAs for normalization augments the reliability of the data produced, and has been advocated by other studies [21], [28].

*MiR-16* appears to be the most widely used EC for blood-related miRNA studies with application to breast, ovarian, pancreatic, gastric, prostate and renal cell cancer, melanoma and the hematological malignancies [9], [14], [25], [30]–[34]. Recent studies have reported on the origin of circulating *miR-16*, indicating red blood cell hemolysis as a major source of this miRNA in blood [35], [36]. This may be more a concern in studies where cell-free blood fragments (serum/plasma) are the source of miRNAs. We utilized whole blood in a disease where it has been previously shown that patient red blood cell and hemoglobin levels are within the normal range in the majority of cases, particularly those with early stage disease [9]. Therefore, as blood samples from both cancer and control patients are treated identically one would not anticipate this to have a direct effect on *miR-16* expression, as evidenced by our results where they was no difference in *miR-16* expression between the cancer and control groups. This issue may be more of a concern, when RNA extraction protocols not utilizing chaotrophic agents such as Trizol are used, implying that individual sample treatment and storage in advance of RNA extraction would directly influence results. There are few reports of *miR-425* in the literature. This is reassuring as it denotes that *miR-425* may be a miRNA with little functional value in disease processes, an attractive trait of an EC. *U6* was selected for further validation by RQ-PCR as although not the most stably expressed EC based on GME or GeNorm analysis of the microarray data, it is commonly used for miRNA studies [11], [12], [37], [38]. *U6* (*RNU6B*) is a small nucleolar RNA (snoRNA) that forms part of the *U6* small nuclear ribonucleoprotein, a component of the spliceosome responsible for splicing of pre-mRNA. The use of *U6* and other snoRNAs in miRNA related research is contentious [39]. These larger molecules are likely to be less reliable than miRNA ECs as their expression is less stable than miRNA with studies showing more frequent degradation in serum samples [40], [41]. This makes it difficult to draw conclusions pertaining to miRNA expression when snoRNAs are utilized as ECs. In this study we showed that *U6* was aberrantly expressed in the cancer group compared to the control group (p = 0.009).

This study focuses on RQ-PCR data normalization using candidate ECs which is the most prevalent method. Two alternative normalization strategies for circulating miRNA expression have been proposed to date; Global Mean Expression (GME) and exogenous (spiked-in) miRNAs. GME was recently introduced by Metsdagh *et al* for use in high-throughput miRNA profiling. GME uses the average expression of all the miRNAs detected in a sample as the normalizer presuming that the mean miRNA expression of all miRNAs is constant when the same starting amount of total RNA is used, regardless of the sample type. This technique reduces technical variation and preserves biological variation and is very suited to large genome wide miRNA profiling [19]. It is better suited to large expression profiling studies, with several such studies reporting its use [42]–[44]. This technique is largely unsuited to biomarker studies as bias may be introduced in such studies when several of the target miRNAs being analyzed show variation in expression (over-expression or under-expression) in one study group compared to another. Spiked-in non-human exogenous miRNAs, such as *cel-miR-39*, *cel-miR-54* and *cel-miR-238*, have also been used for normalization [45]–[47]. This method presumes that by adding a known quantity of spiked in miRNA to an equal volume of serum/plasma/whole blood, a stable quantity of reference gene is obtained. However, this technique leaves room for technical and human error.

Accurate normalization strategies are crucial for miRNA related research, as detecting even small changes in miRNA expression can have major biological implications, as a single miRNA can target multiple mRNAs, even in the same pathway thus augmenting its effect [48]. In truth, a single universal EC for use in all specimen types across all diseases, malignant or otherwise, is unlikely to exist. Suitable ECs need to be validated for use in specific disease states and specimen types. The surge of interest in identifying specific miRNAs as biomarkers for health and disease requires that an equal amount of attention is focused on the establishment of suitable ECs with which to normalize the data such that appropriate conclusions can be derived.

## Conclusion

This study is of relevance in translational miRNA research for circulating miRNAs in breast cancer. It identifies a combination of two miRNAs, *miR-16* and *miR-425*, with application for use as ECs for normalization. Further investigation into suitable ECs for use in miRNA RQ-PCR studies is warranted.
